# Supplementary material for: Mesostructured Fibrils Exfoliated in Deep Eutectic Solvent as Building Blocks of Collagen Membranes
Source: Polymers (Basel). 2023 Oct 6;15(19):4008. doi: 10.3390/polym15194008 (PMC10574992; doi:10.3390/polym15194008)
Supplement: Supplementary file 1 [file polymers-15-04008-s001.zip › polymers-2617479-supplementary.pdf]

Supporting information

Mesostructured Fibrils Exfoliated in Deep Eutectic Solvent as Building Blocks of Collagen Membranes

Ying Pei [1,2,3,*], Wei Li [3], Lu Wang [3], Jing Cui [4], Lu Li [1], Shengjie Ling[4,*], Keyong Tang [3] and Huafeng Tian [2,*]

[1] Key Laboratory of Auxiliary Chemistry and Technology for Chemical Industry, Ministry of Education, Shaanxi University of Science and Technology, Xi'an 710021, China; lilu@sust.edu.cn

[2] Key Laboratory of Processing and Quality Evaluation Technology of Green Plastics of China National Light Industry Council, Beijing Technology and Business University, Beijing 100048, China

[3] College of Materials Science and Engineering, Zhengzhou University, Zhengzhou 450001, China; lw15565152810@126.com (W.L.); 18839150063@163.com (L.W.); kytangzzu@126.com (K.T.)

[4] School of Physical Science and Technology, Shanghai Tech University, Shanghai 201210, China; cuijing@shanghaitech.edu.cn

* Correspondence: peiying@zzu.edu.cn (Y.P.); lingshj@shanghaitech.edu.cn (S.L.); tianhuafeng@th.btbu.edu.cn (H.T.)

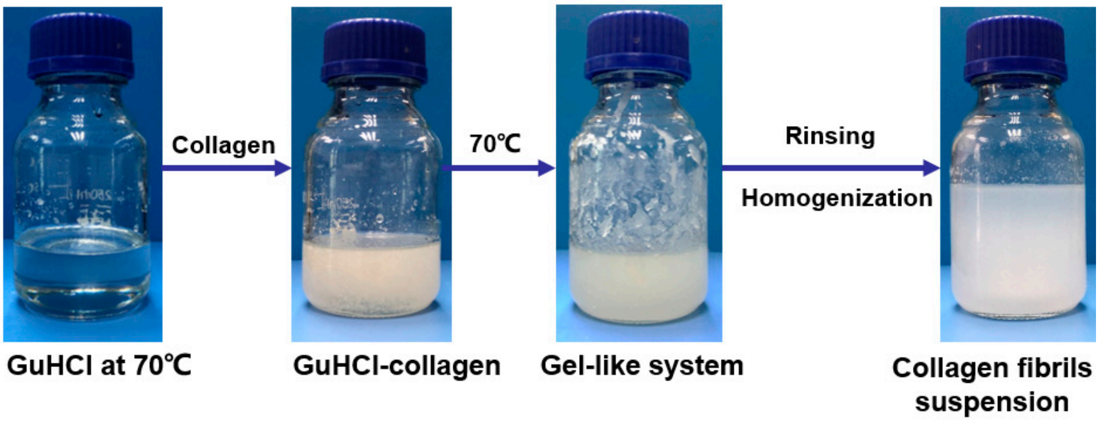

Figure S1 Schematic diagram of the experimental process.

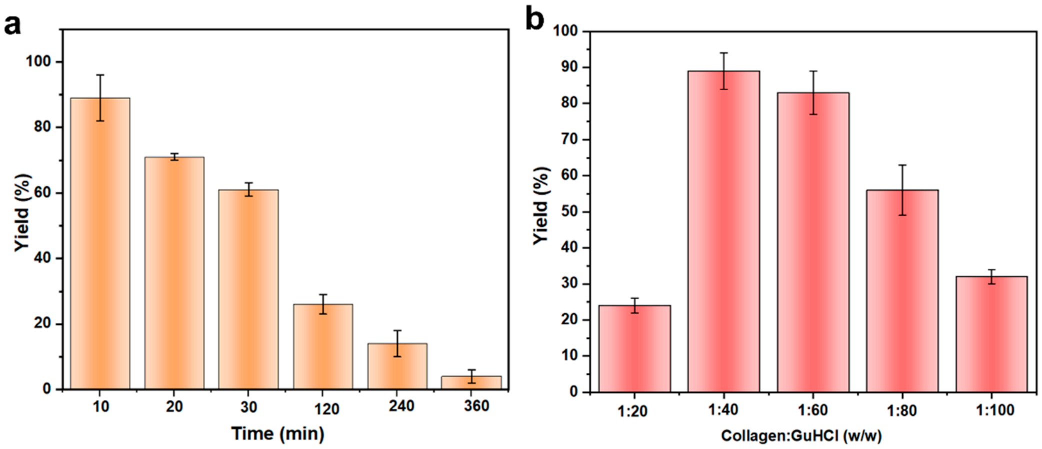

Figure S2 The effects of contacting time (a) and (b) the weight ratio (collagen: GuHCl, w:w) on the yield of collagen fibrils.

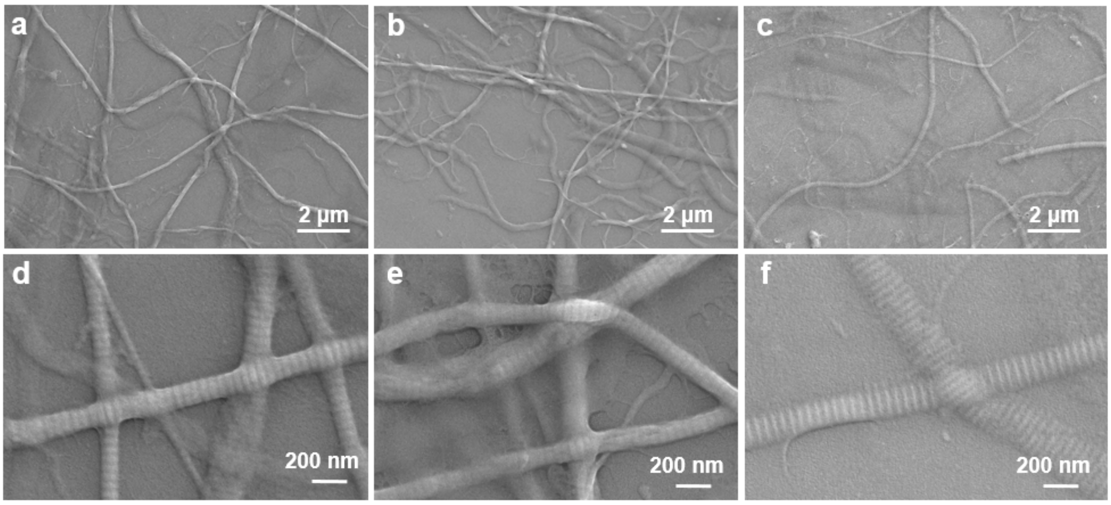

Figure S3 SEM images of collagen fibrils after homogenization for 5 (a, d), 20 (b, e) and 30 (c, f) times.

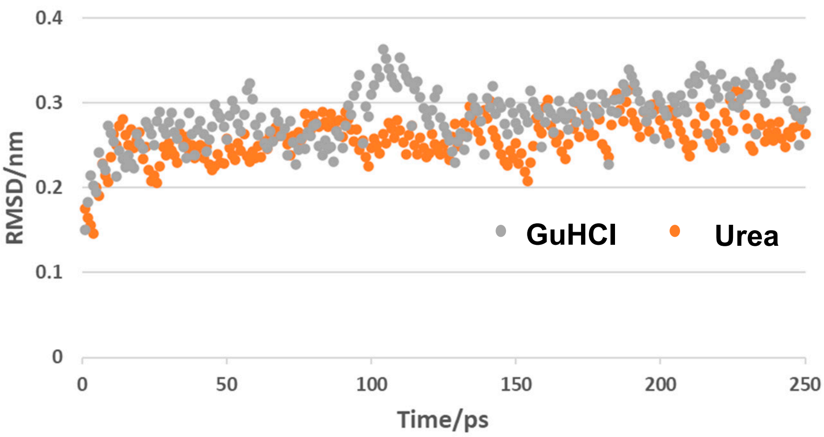

Figure S4 Effects of GuHCl and urea on the root mean square deviation (RMSD) of collagen molecules as a function of simulation time.

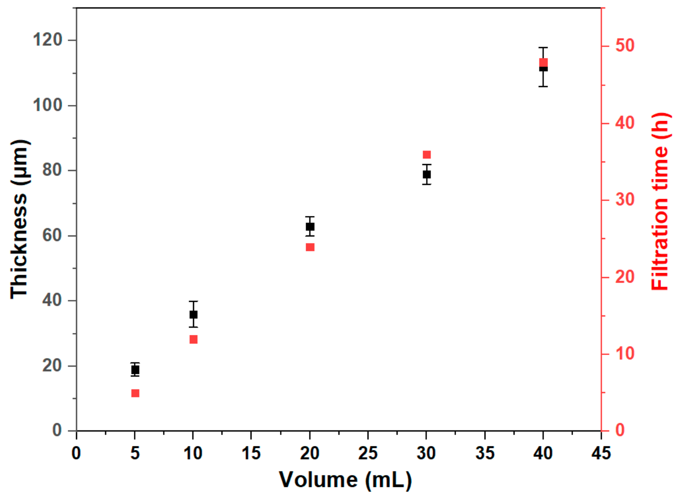

Figure S5 Dependence of CFs suspension volumes on the thickness of collagen membranes and filtration time.

Table S1 Diameter, length and aspect ratio of collagen fibrils by SEM Morphology analysis

Sample	Diameter (nm)	Length (μm)	Aspect ratio
CFs-5	145.23±14.25	62.31±11.30	320-557
CFs-20	123.15±6.55	34.31±8.30	201-358
CFs-30	112.44±9.86	15.74±6.30	74-203

Table S2 Average mechanical data of dry and hydrated collagen membranes.

Sample	Dry state		Hydrated state	
	Ultimate stress (MPa)	Ultimate strain (%)	Ultimate stress (kPa)	Ultimate strain (%)
M-5	70.4±0.9	20±7	270.2±14. 2	102±9
M-20	87.6±1.7	15±5	80.3±5. 3	68±3. 2
M-30	112.5±2.5	9±4	27.6±4. 4	40±4. 9
Control	45.1±1.5	27±3	450±13. 2	17±5. 1

Table S3 The tensile ultimate stress of reported collagen membranes and this work samples

Initial object	Processing methods	Ultimate stress (dry state, MPa)	Ultimate stress (hydrated state, kPa)	Ref.
Collagen solution	Casting	10.0 ± 0.50		[49]
Collagen solution	Casting	49.2 ± 7.10		[50]
Collagen solution	Casting		100±3.00	[51]
Collagen solution	Filtration		1.49±0.90	[52]
Collagen fibrils suspension	Filtration	59.1~111	20~450	This work
